# Contribution of small and medium enterprises to economic development: Evidence from a transiting economy

**DOI:** 10.1016/j.dib.2018.03.126

**Published:** 2018-03-30

**Authors:** James Obi, Ayodotun Stephen Ibidunni, Atolagbe Tolulope, Maxwell Ayodele Olokundun, Augusta Bosede Amaihian, Taiye Tairat Borishade, Peter Fred

**Affiliations:** Department of Business Management, Covenant University, Ota, Ogun State, Nigeria

**Keywords:** Entrepreneurship, SMEs, Economic development, Transiting economy, Nigeria

## Abstract

The focus of this research was to present a data article on the contribution of SMEs to economic development in a transiting economy. Descriptive research design was adopted in this study. Data were obtained from 600 respondents in 60 small-scale enterprises located in different parts of the country (20 small-scale enterprises located in Lagos State, 20 in Anambra State and 20 in Kano State of Nigeria respectively). Data analysis was carried out using tables and percentages and the null hypotheses of the study was tested using chi-square (*X*^2^) inferential statistical model at 5% level of significance. The findings revealed that there is a significant relationship between the operation of small and medium-scale enterprises and economic growth in developing nations.

## Specifications Table

**Table:** 

Subject area	*Entrepreneurship, Economy*
More specific subject area	*Small and Medium Enterprises, Economic Development*
Type of data	*Table, figure*
How data was acquired	*Researcher made questionnaire analysis*
Data format	*Raw, analyzed, descriptive and statistical data*
Experimental factors	- *Samples consist of owner/managers of SMEs in three major commercial states of Nigeria*. - *In this paper, the strategic contributions of SMEs to job creation, poverty alleviation and standard of living of people was studied.*
Experimental features	*SMEs are critical to economic development, especially of transiting economies*
Data source location	*SMEs in Lagos, Anambra and Kano States of Nigeria*
Data accessibility	*Data is included in this article*

## Value of the data

• These data describe demographic data of SME owner/managers across three strategic industrial sectors in Nigeria, in order to understand the influence of their background factors to issues relating to SMEs’ contribution to economic development.
• The data showed that SMEs contributions are very essential to improving economic development especially in the areas of job creation, poverty alleviation and standard of living of people. These aspects of economic development are very strategic to transiting economies, like Nigeria [1], [4].
• Considering their strategic roles to promoting economic value, the data from this study can be used by policy makers and researchers to understand the importance of SMEs [2], [3], [7], especially in the agricultural, manufacturing and trading sectors, towards the attainment of economic development.

## 1 Data

In the distribution of respondents by gender; 380 (63.3%) were male, while 220 (36.7%) were female. This shows that a larger percentage of the men are participating in small-scale business in the areas studied (Table 1).

**Table 1:** Gender classification of the respondents.

**Gender**	**Number of respondents (Frequency)**	**Percentage**
Male	380	63.3%
Female	220	36.7%
**Total**	**600**	**100%**

Table 2 shows the distribution of respondents by age and educational qualification. It can be seen that greater number of people participating in small-scale business fall within the age bracket of 30–50 (53.4%). These are matured people in life. However, youths are also taking more and more interest in small-scale business since they rank second at 200 (33.3%).

**Table 2:** Classification of respondents by age and educational qualification.

**Age**			**Educational qualification**		
	**Frequency**	**Percentage**		**Frequency**	**Percentage**
18–30	200	33.3%	Primary school	120	20%
30–50	320	53.4%	Secondary School	400	66.7%
50–Above	80	13.3%	Tertiary institution	80	13.3%
	**600**	**100**		**600**	**100**

Majority of the people that are participating in small-scale enterprises in the study are within the secondary school level numbering 400 (66.7%). They are generally young people trying to stand on their own by embarking on small-scale business rather than looking for jobs in the job market.

Table 3 shows that majority of the small-scale enterprises are still relatively new having been in business for less than 5 years showing a figure of 240 (49%). Furthermore, majority of the people in small-scale businesses engage in trading and other artisan work showing a figure of 334 (55.7%). Fewer number of small-businesses owners are in manufacturing and agriculture.

**Table 3:** Distribution of respondents by year spent in business and nature of business.

**Years spent in business**			**Nature of business**		
	**Frequency**	**Percentage**		**Frequency**	**Percentage**
1–5 years	240	40%	Agriculture	100	16.6%
5–10 years	185	30.8%	Manufacturing	166	27.7%
10 yrs. and Above	175	29.2%	Trading & others	334	55.7%
	**600**	**100**		**600**	**100**

### 1.1 Testing of hypothesis and results

Results of the study were presented in line with the three formulated hypotheses as given below:

Ho_1_ Small-scale enterprises are not significantly contributing to job creation.

Table 4 shows that *X*^2^ calculated value (860.6) is greater than *X*^2^ table value (9.49), i.e. *X*^2^c > *X*^2^t at 5% (0.05) level of significance. The Null hypothesis (H_o_) is rejected. The result of the hypothesis tested revealed that there is positive and significant relationship between the operation of the small-scale enterprises and job creation. In other words, small-scale enterprises are making significant impact on job creation. This finding supports the claims of [5] as perceiving SMEs to be fundamental to supporting job creation drive of a nation.

Ho_2_ Small-scale enterprises are not making significant impact on poverty alleviation.

**Table 4:** Chi-square test measured the relationship between small-scale enterprises operation and job creation.

Item	*N*	D*f* (v)	*X*^2^c	*X*^2^t	Significance
Strongly Agree	366	4	860.6	9.49	Significant
Agree	204				
Undecided	10				
Disagree	8				
Strongly Disagree	12				
*P* < 0.05	600				

Table 5 shows that *X*^2^ calculated value (438.4) is greater than *X*^2^ table value (9.49), i.e. *X*^2^c > *X*^2^t at 0.05 level of significance. The Null hypothesis (H_o_) is rejected. The result of the hypothesis tested revealed that small-scale enterprises are making significant impact on poverty alleviation in the country through employment opportunities they provide to the people [6]. There is significant relationship between small-scale enterprises activities and poverty alleviation.

Ho_3_ The operation of small-scale enterprises is not significantly contributing to improvement in the standard of living.

**Table 5:** Chi-square test measures the relationship between small-scale enterprises activities and poverty alleviation.

Item	*N*	D*f* (v)	*X*^2^c	*X*^2^t	Significance
Strongly Agree	280	4	438.4	9.49	Significant
Agree	200				
Undecided	68				
Disagree	37				
Strongly Disagree	15				
*P* < 0.05	600				

Table 6 shows that *X*^2^ calculated value (564.9) is greater than *X*^2^ table value (9.49), i.e. *X*^2^c > *X*^2^t at 0.05 level of significance. The Null hypothesis (H_o_) is rejected. The result of the hypothesis tested revealed that the operation of small-scale enterprises is significantly contributing to improvement in the standard of living of the people. This implies that there is significant positive relationship between small-scale enterprises operation and improvement in the standard of living.

**Table 6:** Chi-square test measured the relationship between small-scale enterprises operation and improvement in the standard of living of the people.

Item	*N*	D*f* (v)	*X*^2^c	*X*^2^t	Significance
Strongly Agree	305	4	564.9	9.49	Significant
Agree	211				
Undecided	41				
Disagree	22				
Strongly Disagree	21				
*P* < 0.05	600				

## 2 Experimental design, materials and methods

The researcher adopted survey research design to obtain data from 600 respondents from 60 small-scale enterprises. These 60 SMEs were distributed as: 20 small-scale enterprises were located in Lagos State, 20 in Anambra State and 20 in Kano State of Nigeria respectively. Data was gathered by means of structured questionnaire. The questionnaire was divided into sections A and B. Section A was used to obtain demographic information from respondents. Section B assessed the contribution of small-scale enterprises to economic development. Three null hypotheses were formulated to guide the analysis of data. Data obtained were analyzed using Tables, percentages and chi-square (*X*^2^) test at 5% level of significance. Ethical consideration in the research process was ensured because administering the questionnaires to respondents was based on their willingness to respond to the research instrument. Moreover, confidentiality and anonymity for participants in the study was assured.

## 3 Conclusion and implications of the study

The evidences from the data presented point to the fact that small and medium enterprises are significant driver of economic development, especially in transition economies like Nigeria. This has significant implications for practice because government of such economies must focus on initiating programmes that encourage the creation of SMEs, while also motivating existing SMEs to sustain performance and growth. More so, the data presented in this article is significant to guiding further investigations in extensive research.
