# Supplementary material for: Contribution of small and medium enterprises to economic development: Evidence from a transiting economy
Source: Data Brief. 2018 Mar 30;18:835–9. doi: 10.1016/j.dib.2018.03.126 (PMC5996597; doi:10.1016/j.dib.2018.03.126)
Supplement: Supplementary file 1 — Supplementary material [file mmc1.doc]

Ibidunni, Ayodotun Stephen (PhD)

Covenant University,

Ota,

Ogun State,

Nigeria

21st March, 2018

The Editor,

Data In Brief,

Dear Sir,

**DECLARATION OF CONFLICT OF INTEREST**

I, Dr. Ibidunni, Ayodotun Stephen and my colleagues write to declare that there is no conflict of interest traceable to our data paper “***Contribution of Small and Medium Enterprises to Economic Development: Evidence from a Transiting Economy***”

Yours faithfully,


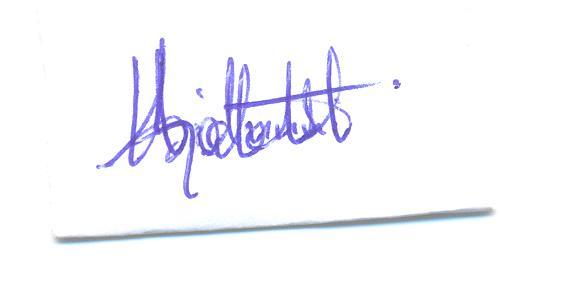


Ibidunni, Ayodotun Stephen (PhD) (Corresponding Author)

+234-803-489-3637
